# Cloud-Edge Resource Scheduling and Offloading Optimization Based on Deep Reinforcement Learning

**DOI:** 10.3390/s26051704

**Published:** 2026-03-08

**Authors:** Lili Yin, Yunze Xie, Ze Zhao, Jie Gao

**Affiliations:** School of Computer Science and Technology, Harbin University of Science and Technology, Harbin 150080, China; 2320410142@stu.hrbust.edu.cn (Y.X.); 2420410178@stu.hrbust.edu.cn (Z.Z.); 2420410126@stu.hrbust.edu.cn (J.G.)

**Keywords:** task offloading, resource scheduling, convolutional neural networks, informer, deep reinforcement learning, Deep Q-Networks

## Abstract

In the context of smart manufacturing, with the widespread deployment of Industrial Internet of Things (IoT) devices, a large number of computation tasks that are highly sensitive to latency and have strict deadlines have emerged, requiring real-time processing. Effectively offloading tasks to address the issues of increased latency and task dropouts caused by dynamic changes in edge node load has become a key challenge in the cloud–edge–end collaborative environment of smart manufacturing. To tackle the complex issues of unknown edge node loads and dynamic system state changes, this paper proposes a distributed algorithm based on deep reinforcement learning, utilizing convolutional neural networks (CNN) and the Informer architecture. The proposed algorithm leverages CNN to extract local features of edge node loads while utilizing Informer’s self-attention mechanism to capture long-term load variation trends, thereby effectively handling the uncertainty and dynamics inherent in node loads. Furthermore, by integrating the Dueling Deep Q-Network (DQN) and Double DQN techniques, the algorithm achieves a precise approximation of the state–action value function, further enhancing its capability to perceive system temporal characteristics and adapt to heterogeneous tasks. Each mobile device can independently make task offloading decisions and scheduling strategies based on its observations, enabling dynamic task allocation and optimization of execution order. Simulation results show that, compared to various existing algorithms, the proposed method reduces task dropout rates by 82.3–94% and average latency by 28–39.2%. Experimental results validate the significant advantages of this method in intelligent manufacturing scenarios with high load and latency-sensitive tasks.

## 1. Introduction

Industry 5.0 is an advanced and evolved smart manufacturing system built upon Industry 4.0, emphasizing a “human-centric” design philosophy [1]. It effectively integrates a cloud–edge–end collaborative architecture to meet the modern manufacturing industry’s demands for high reliability, low latency, and flexible customization [2]. Within this architecture, the cloud platform is responsible for large-scale historical data storage, deep model training, and global resource scheduling [3]. Edge nodes are deployed near production sites to perform near-source processing for latency-sensitive tasks, such as online quality inspection and fault warning. The terminal side consists of various sensors and industrial robots, which are responsible for real-time data collection and instruction execution, thereby achieving end-to-end closed-loop control and automated optimization [4]. Industrial robots serve as critical execution units within the system, enabling precise processing, collaborative operations, and flexible scheduling of production processes through cloud–edge–end collaboration. By leveraging real-time sensing and feedback mechanisms, they continuously optimize motion trajectories and operational parameters, thereby enhancing overall production efficiency and system robustness [5]. While improving efficiency and safety, challenges such as bandwidth bottlenecks, limited edge computing resources, system stability, and cost control remain [6]. To address these challenges, Mobile Edge Computing (MEC) deploys distributed computing and storage resources at the network edge, offloading certain computational tasks from centralized cloud environments to the edge and endpoints, thereby effectively managing task scheduling and offloading [7]. Task scheduling involves allocating tasks across multiple available resources to maximize system performance and resource utilization [8]. It requires precise decision-making across different resources (such as cloud and edge devices) to ensure that tasks are completed on time and processed in the shortest possible time. To optimize task scheduling strategies, factors such as resource distribution, task execution timeliness, computational requirements, network bandwidth, system load, and resource availability must also be comprehensively considered [9]. In edge computing environments, due to limitations in network bandwidth and computational capabilities, task scheduling must balance resource allocation between the cloud and edge to avoid bandwidth bottlenecks or over-reliance on edge computing resources, thus improving overall system performance and response speed [10]. When mobile or edge devices are constrained, computation-intensive or storage-intensive tasks can be offloaded to cloud or edge servers for execution. This not only alleviates network bandwidth and latency pressures but also enhances the system’s real-time response capability and robustness, laying the foundation for efficient collaboration in smart manufacturing scenarios [11].

In a smart manufacturing environment, a large number of terminal devices simultaneously offload tasks to mobile edge computing (MEC) nodes, which can lead to a significant increase in node load and task processing latency and even result in tasks being discarded. To address this challenge, various task offloading strategies have been proposed in the literature, which can be broadly categorized into four methodological streams: heuristic-based approaches, centralized deep reinforcement learning, distributed DRL frameworks, and prediction-assisted scheduling methods.

- Heuristic-Based Approaches

Early efforts focused on heuristic algorithms that leverage local system information to make offloading decisions. Huang et al. [12] proposed a cloud–edge collaboration solution that uses service orchestration to meet the demands of low-latency, high-reliability applications. Suganya et al. [13] designed the DTOE-AOF framework, which dynamically estimates node load and communication latency to optimize task offloading in unmanned aerial vehicle (UAV) applications. Chen et al. [14] proposed a multi-hop task offloading model to accelerate the execution of complex tasks. Wu et al. [15] introduced a latency-aware and energy-efficient industrial IoT task offloading method. While these heuristic approaches are computationally lightweight, they typically rely on simplified system models and lack adaptability to dynamic load fluctuations, often leading to suboptimal performance under rapidly changing edge conditions.

B. Centralized Deep Reinforcement Learning Methods

To enhance adaptability, recent studies have adopted deep reinforcement learning for task offloading in MEC environments. Yamansavascilar et al. [16] proposed the DeepEdge framework, which uses a central coordinator to collect global state information and employs DRL to make offloading decisions, effectively improving task completion rates and latency performance. Huang et al. [17] proposed an online optimization scheme for DRL in wireless power supply scenarios, addressing energy consumption and latency issues. Wu et al. [18] investigated DRL-based online task offloading, demonstrating its effectiveness in adapting to time-varying network dynamics. However, centralized DRL methods require global state information and a central coordinator, which limits their scalability in large-scale industrial deployments and introduces single points of failure.

C. Distributed DRL Frameworks

To overcome scalability limitations, distributed DRL approaches have emerged where each terminal device makes independent offloading decisions based on local observations. Tang et al. [19] proposed a distributed DRL algorithm to address uncertain edge node loads. Sun et al. [20] introduced a hierarchical DRL method to jointly optimize service caching and computation offloading. Huang et al. [21] utilized partially observable DRL for deadline-aware task offloading. Gao et al. [22] employed an attention-based multi-agent DRL algorithm for resource allocation in heterogeneous networks. Gong et al. [23] developed a DRL architecture tailored for industrial IoT, jointly optimizing task offloading and resource allocation. Despite their improved scalability, these distributed methods either rely on single-scale feature extraction, making it difficult to balance short-term fluctuations with long-term trends, or introduce additional coordination overhead that complicates real-time decision-making.

D. Prediction-Assisted Scheduling

Parallel to DRL-based methods, research on prediction-driven resource scheduling has demonstrated that leveraging deep time-series models for prior prediction of edge load enhances scheduling robustness. Gao et al. [24] proposed integrating Conv1D, LSTM, and GRU for time-series prediction in edge resource scheduling, validating its effectiveness on a self-built dataset. Qin et al. [25] proposed a prediction-assisted DRL framework for MEC task offloading, where historical system states are exploited to estimate future resource availability. Zhao et al. [26] introduced a graph attention-based reinforcement learning approach for congestion-aware task offloading. These studies highlight the importance of both short-term and long-term sequence information for scheduling decisions, motivating the multi-scale temporal modeling approach adopted in this paper.

To provide a clear overview of the existing literature and highlight the research gap addressed in this paper, Table 1 summarizes the key characteristics of the main methodological categories discussed above.

As shown in Table 1, existing methods exhibit distinct trade-offs between scalability, load perception capability, and coordination overhead. Heuristic approaches offer high scalability but lack adaptability to dynamic conditions. Centralized DRL methods achieve better adaptability but at the cost of scalability. Distributed DRL frameworks improve scalability but typically focus on single-scale feature extraction, neglecting the interplay between short-term load bursts and long-term trends. Prediction-assisted methods incorporate historical data but primarily focus on short-term predictions. To address these limitations, this paper proposes a distributed DRL algorithm that combines CNN-based short-term feature extraction with Informer-based long-term trend modeling, achieving multi-scale load perception while maintaining low coordination overhead and high real-time suitability.

Despite the progress made by existing approaches, three key limitations persist: centralized methods lack scalability; distributed methods rely on single-scale feature extraction, failing to capture both short-term bursts and long-term load trends; and prediction-assisted methods primarily focus on short-term forecasting without modeling long-range dependencies. To address these gaps, this paper proposes a distributed deep reinforcement learning task offloading algorithm based on a hybrid CNN-Informer architecture, with Dueling Double DQN (D3QN) serving as the decision algorithm. CNN is used to efficiently extract local features of short-term load fluctuations at edge nodes, while Informer models long-term trends and temporal dependencies through self-attention mechanisms. The combination of the two enables multi-scale feature perception, allowing each mobile device to make real-time, adaptive offloading decisions independently. Experimental results demonstrate that, compared to existing algorithms, our method reduces task dropout rates by 82.3–94% and average latency by 28–39.2%, thereby significantly enhancing system performance in complex intelligent manufacturing scenarios.

## 2. System Model

**Figure 1:**
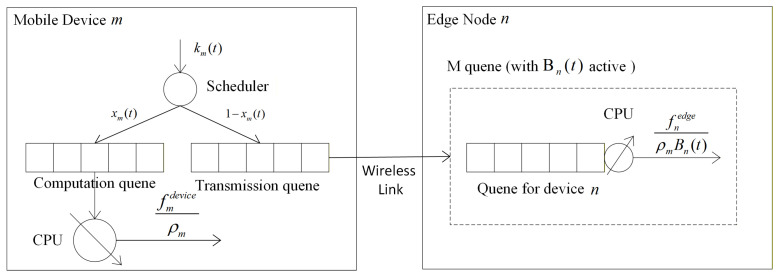
MEC System Model Diagram.

Consider a mobile edge computing (MEC) system comprising a set of edge nodes, denoted by N={1,2,…,N}, and a set of mobile terminal devices, denoted by M={1,2,…,M}. Each mobile device can offload its computational tasks to the edge nodes for processing. The system operates in a discrete-time framework, where time is divided into slots of equal duration Δ seconds, indexed by the set T={1,2,…,T}. The modeling of mobile devices and edge nodes will be detailed in the following subsections, and the overall system architecture is depicted in Figure 1.

### 2.1. Terminal Device Model

We adopt a discrete-time model with slot duration Δ=0.1 s, ensuring that at most one new task arrives per device per time slot. Each mobile device m∈M is equipped with a scheduling unit that, at the beginning of each time slot t∈T, classifies newly arrived tasks. If a task is designated for local execution, it enters the local computation queue; otherwise, it is placed in the transmission queue for offloading to an edge server via the wireless link (see Figure 1). We assume that if a task completes its computation or transmission within the current time slot, the next queued task starts processing immediately in the subsequent slot. The negligible overhead introduced by slot switching is ignored when tasks experience extended queuing delays. The following subsections detail the task model, offloading decision strategy, local computation queue, and transmission queue.

Unit convention: CPU and wireless link capacities are specified as per-second rates (e.g., GHz, Mbps). When computing the number of required time slots, we multiply these rates by the slot length Δ to obtain per-slot capacities. Concretely, the per-slot CPU capacities are $f_{m}^{\mathrm{device}}\Delta$ and $f_{n}^{\mathrm{edge}}\Delta$ (in cycles/slot), and the per-slot transmission capacity is $f_{m,n}^{\mathrm{tran}}\Delta$ (in bits/slot). Task size $\lambda_{m}\left(t\right)$ is in bits, processing density $\rho_{m}$ in cycles/bit, and deadlines $\tau_{m}$ in time slots.

#### 2.1.1. Task Model

At the beginning of each time slot t∈T, a mobile device m∈M may receive a new task. We define the variable $k_{m}\left(t\right)\in Z_{++}$ to represent the unique ID of the task, and let $\lambda_{m}\left(t\right)$ represent the size of the new task data (in bits). If a new task $k_{m}\left(t\right)$ arrives at the start of time slot *t*, then $\lambda_{m}\left(t\right)$ equals the size of that task; if no new task arrives during that time slot, then both $k_{m}\left(t\right)$ and $\lambda_{m}\left(t\right)$ are zero. The task size $\lambda_{m}\left(t\right)$ takes values from a discrete set $\Lambda ≜\{\lambda_{1},\lambda_{2},\ldots ,\lambda_{\left|\Lambda \right|}\}$, where |Λ| denotes the number of available sizes. Therefore, $\lambda_{m}\left(t\right)\in \Lambda \cup \left\{0\right\}$. If no task arrives at time slot *t*, then $k_{m}\left(t\right)=0$ and $\lambda_{m}\left(t\right)=0$. A task is considered failed and discarded if it is not fully processed by the end of time slot $t+\tau_{m}-1$.

#### 2.1.2. Task Offloading Decision

When time slot *t* begins, if device *m* captures a new task to be processed, we need to decide between local processing and wireless offloading; otherwise, no offloading operation is performed during that time slot. This paper introduces a set of binary decision variables to characterize the choice between local and remote processing of tasks, as well as offloading decisions among edge nodes. Let the binary variable $x_{m}\left(t\right)\in \left\{0,1\right\}$ indicate whether task $k_{m}\left(t\right)$ is scheduled to the computation queue or the transmission queue. If $x_{m}\left(t\right)=1$, the task is scheduled to the local computation queue; if $x_{m}\left(t\right)=0$, it is scheduled to the transmission queue for offloading. Next, we define a binary variable $y_{m,n}\left(t\right)\in \left\{0,1\right\}$ to indicate whether task $k_{m}\left(t\right)$ is offloaded to edge node n∈N. If $y_{m,n}\left(t\right)=1$, the task is offloaded to edge node *n*; if $y_{m,n}\left(t\right)=0$, it is not offloaded to that node. For notational convenience, we define the vector $y_{m}\left(t\right)={\left(y_{m,n}\left(t\right)\right)}_{n\in N}$. To ensure that each task is offloaded to exactly one edge node, thereby simplifying scheduling logic and improving execution efficiency, the following constraint applies:

(1) $$\sum_{n\in N}y_{m,n}\left(t\right)=I\left\{x_{m}\left(t\right)=0\right\},\forall m\in M,\;t\in T$$

where I{z∈Z} is the indicator function, which equals 1 if the condition inside the braces is true, and 0 otherwise.

#### 2.1.3. Computation Queue

In mobile edge computing systems, to describe the queuing and processing flow of terminals during local computing, we introduce the following concepts and calculation steps. Let $f_{m}^{\mathrm{device}}$ (unit: cycles/second) represent the processing capacity of device *m* per second. Therefore, the per-slot processing capacity is $f_{m}^{\mathrm{device}}\Delta$ (cycles/slot). Considering the processing density $\rho_{m}$ (cycles/bit), device *m* can process at most $\left(f_{m}^{\mathrm{device}}\Delta \right)/\rho_{m}$ bits of data in a time slot. When time slot *t* begins, if task $k_{m}\left(t\right)$ has been added to the local computation queue, we use $l_{m}^{\mathrm{comp}}\left(t\right)$ to denote the time slot at which the task is actually completed (or discarded due to exceeding the deadline). If task $k_{m}\left(t\right)$ is not placed in the computation queue, or if the task ID $k_{m}\left(t\right)=0$, then $l_{m}^{\mathrm{comp}}\left(t\right)$ is uniformly set to 0. Let $\delta_{m}^{\mathrm{comp}}\left(t\right)$ denote the number of time slots that task $k_{m}\left(t\right)$ must wait after being placed in the computation queue before being processed. For any m∈M, t∈T, the calculation of $\delta_{m}^{\mathrm{comp}}\left(t\right)$ is as follows:

(2) $$\delta_{m}^{\mathrm{comp}}\left(t\right)=\max_{t^{\prime }\in \left\{0,1,\ldots ,t-1\right\}}{\left[l_{m}^{\mathrm{comp}}\left(t^{\prime }\right)-t+1\right]}^{+}$$

Specifically, the term $\max l_{m}^{\mathrm{comp}}\left(t^{\prime }\right)$ represents the latest time slot in which all tasks that have been placed in the computation queue before the current time slot *t* were processed or discarded. If mobile device m∈M places task $k_{m}\left(t\right)$ into the computation queue at the start of time slot t∈T ($x_{m}\left(t\right)=1$), $l_{m}^{\mathrm{comp}}\left(t\right)$ indicates that task $k_{m}\left(t\right)$ was either completed before this time slot or discarded after this time slot due to timeout. The formula for calculating $l_{m}^{\mathrm{comp}}\left(t\right)$ is as follows:

(3) $$l_{m}^{\mathrm{comp}}\left(t\right)=\min\left\{t+\delta_{m}^{\mathrm{comp}}\left(t\right)+\left\lceil \frac{\lambda_{m}\left(t\right)}{\left(f_{m}^{\mathrm{device}}\Delta \right)/\rho_{m}}\right\rceil -1,\;t+\tau_{m}-1\right\}$$

Equation (3) indicates that the actual completion time is determined by the sum of waiting time and service time, truncated by the task deadline constraint.

#### 2.1.4. Transmission Queue

To describe the queuing and completion of data transmission from mobile terminals to edge nodes, let $f_{m}^{\mathrm{tran}}$ (bits/slot) denote the maximum available wireless transmission rate for device *m* in each time slot. If task $k_{m}\left(t\right)$ is selected for the transmission queue at the start of time slot *t* (i.e., the offloading decision is remote), then let $l_{m}^{\mathrm{tran}}\left(t\right)$ denote the time slot at which the task is ultimately completed or timed out and discarded. If no task is added to the transmission queue in that time slot, or if $k_{m}\left(t\right)=0$, then $l_{m}^{\mathrm{tran}}\left(t\right)$ is uniformly set to 0. Let $\delta_{m}^{\mathrm{tran}}\left(t\right)$ (units: time slots) denote the number of time slots a task $k_{m}\left(t\right)$ must wait before transmission begins, if it is added to the transmission queue. For any m∈M, t∈T, the formula for calculating $\delta_{m}^{\mathrm{tran}}\left(t\right)$ is as follows

(4) $$\delta_{m}^{\mathrm{tran}}\left(t\right)=\max_{t^{\prime }\in \left\{0,1,\ldots ,t-1\right\}}{\left[l_{m}^{\mathrm{tran}}\left(t^{\prime }\right)-t+1\right]}^{+}$$

The transmission completion time follows the same queuing logic as the local computation case, subject to deadline truncation.

(5) $$l_{m}^{\mathrm{tran}}\left(t\right)=\min\left\{t+\delta_{m}^{\mathrm{tran}}\left(t\right)+\left\lceil \frac{\sum_{n\in N}y_{m,n}\left(t\right)\lambda_{m}\left(t\right)}{f_{m,n}^{\mathrm{tran}}\Delta }\right\rceil -1,\;t+\tau_{m}-1\right\}$$

The waiting time for the transmission queue is analogous to that of the computation queue, taking the smaller value between the “time required for completion” and the “deadline”.

### 2.2. Edge Node Model

In this system, each edge node n∈N is assigned a dedicated queue channel for each mobile device m∈M to receive and cache offloading tasks from that device. When time slot *t* ends, if the offloading data from device *m* has been fully received by node *n*, the task is appended to the end of the queue corresponding to device *m* in time slot t+1. This paper uses the positive integer $k_{mn}^{\mathrm{edge}}\left(t\right)$ to denote the *k*-th task offloaded by device *m* to node *n* in time slot *t* (set to 0 if there are no new tasks). When $k_{mn}^{\mathrm{edge}}\left(t\right)\neq 0$, $\lambda_{mn}^{\mathrm{edge}}\left(t\right)$ represents the data size of the task; otherwise, it is 0. The dynamic nature of queue lengths and processing rates at edge nodes introduces uncertainty in task waiting and processing times. The exact queuing and computation latency can only be inferred after task completion (or timeout discard) when the system reports the “completion time slot”. The following sections detail the task queue model and edge node processing/discard mechanisms.

#### 2.2.1. Edge Node Task Queue Model

At edge node *n*, each mobile device *m* corresponds to an independent queue used to store data offloaded from device *m*. To describe the dynamic evolution of each device-specific queue at the edge node, let $q_{m,n}^{\mathrm{edge}}\left(t\right)$ represent the number of bits of data remaining in the queue of device *m* at node *n* at the end of time slot *t*. If new data arrives in time slot *t* ($\lambda_{m,n}^{\mathrm{edge}}\left(t\right)>0$) or the queue from the previous time slot is not empty ($q_{m,n}^{\mathrm{edge}}\left(t-1\right)>0$), the queue is referred to as an active queue. Collecting the device IDs corresponding to all active queues into the set $B_{n}\left(t\right)$, we have:

(6) $$B_{n}\left(t\right)=\left\{m|m\in M,\lambda_{m,n}^{\mathrm{edge}}\left(t\right)>0\;\mathrm{or}\;q_{m,n}^{\mathrm{edge}}\left(t-1\right)>0\right\}$$

Let $|B_{n}\left(t\right)|$ denote the number of active queues, and each edge node has $f_{n}^{\mathrm{edge}}$ CPU cycles per second. According to the generalized GPS principle, the $|B_{n}\left(t\right)|$ active queues share the node’s processing capacity. Considering the processing density per bit $\rho_{m}$ (cycles/bit), each active queue can process at most $\frac{f_{n}^{\mathrm{edge}}\Delta }{\rho_{m}\left|B_{n}\left(t\right)\right|}$ bits in this time slot.

Let $q_{m,n}^{\mathrm{edge}}\left(t\right)$ be the remaining data volume at the end of time slot *t*, accounting for the newly arrived data $\lambda_{m,n}^{\mathrm{edge}}\left(t\right)$, subtracting the data processed in this time slot or discarded due to timeout $e_{m,n}^{\mathrm{edge}}\left(t\right)$, and taking non-negative values. This leads to the following queue length update equation:

(7) $$q_{m,n}^{\mathrm{edge}}\left(t\right)={\left[q_{m,n}^{\mathrm{edge}}\left(t-1\right)+\lambda_{m,n}^{\mathrm{edge}}\left(t\right)-\frac{f_{n}^{\mathrm{edge}}\Delta }{\rho_{m}\left|B_{n}\left(t\right)\right|}\cdot I\left\{m\in B_{n}\left(t\right)\right\}-e_{m,n}^{\mathrm{edge}}\left(t\right)\right]}^{+}$$

This model fully describes the reception, allocation, processing, and discarding dynamics of each queue in each time slot.

#### 2.2.2. Edge Node Task Processing and Discard Model

To describe the processing time slots and discard mechanism for tasks at edge nodes, define ${\hat{t}}_{m,n}^{\mathrm{edge}}\left(t\right)$ as the time slot when the task actually begins processing, ensuring that it is neither earlier than the arrival time slot nor earlier than the next time slot corresponding to the completion or discard of all previous tasks in the queue. For any m∈M, n∈N, t∈T, the following holds:

(8) $${\hat{t}}_{m,n}^{\mathrm{edge}}\left(t\right)=\max\left\{t,\max_{t^{\prime }\in \left\{0,1,\ldots ,t-1\right\}}t_{m,n}^{\mathrm{edge}}\left(t^{\prime }\right)+1\right\}$$

Let $t_{m,n}^{\mathrm{edge}}\left(t\right)$ denote the time slot when the task offloaded by mobile device *m* to edge node *n* at time slot *t* is either “completed” or “forcibly discarded.” Given the load state of the edge node, the smallest time slot satisfying the following constraints is determined. For any m∈M, n∈N, t∈T, the following applies:

(9) $$\sum_{t^{\prime }={\hat{t}}_{m,n}^{\mathrm{edge}}\left(t\right)}^{t_{m,n}^{\mathrm{edge}}\left(t\right)}\frac{f_{n}^{\mathrm{edge}}\Delta }{\rho_{m}\left|B_{n}\left(t^{\prime }\right)\right|}\cdot I\left\{m\in B_{n}\left(t^{\prime }\right)\right\}\geq \lambda_{m,n}^{\mathrm{edge}}\left(t\right)$$

(10) $$\sum_{t^{\prime }={\hat{t}}_{m,n}^{\mathrm{edge}}\left(t\right)}^{t_{m,n}^{\mathrm{edge}}\left(t\right)-1}\frac{f_{n}^{\mathrm{edge}}\Delta }{\rho_{m}\left|B_{n}\left(t^{\prime }\right)\right|}\cdot I\left\{m\in B_{n}\left(t^{\prime }\right)\right\}<\lambda_{m,n}^{\mathrm{edge}}\left(t\right)$$

The transmission completion time follows the same queuing logic as the local computation case, subject to deadline truncation.

## 3. Task Offloading in Mobile Computing

In a mobile edge computing environment, terminal devices must decide at the beginning of each time slot whether newly generated tasks should be processed locally or offloaded to edge nodes, based on their observed operating status (including the size of newly arrived task data, local and edge queue lengths, etc.). When a task is successfully completed, its delay cost is recorded. If the task cannot be completed due to resource constraints or timeouts, a corresponding task discard penalty is incurred. Devices must establish a state-action mapping decision strategy with the optimization goal of minimizing the overall average cost in the long term.

### 3.1. State Representation

At the beginning of each time slot *t*, terminal device *m* aggregates the information it observes into a state vector $s_{m}\left(t\right)$. This state vector is defined as follows:

(11) $$s_{m}\left(t\right)=\left(\lambda_{m}\left(t\right),\delta_{m}^{\mathrm{comp}}\left(t\right),\delta_{m}^{\mathrm{tran}}\left(t\right),q_{m}^{\mathrm{edge}}\left(t-1\right),H\left(t\right)\right)$$

Specifically, this vector consists of the following components: $\lambda_{m}\left(t\right)$: The data size (in bits) of new tasks arriving in the time slot; $\delta_{m}^{\mathrm{comp}}\left(t\right)$: The number of time slots a task must wait if scheduled into the local computation queue; $\delta_{m}^{\mathrm{tran}}\left(t\right)$: The number of time slots a task must wait in the transmission queue; $q_{m}^{\mathrm{edge}}\left(t-1\right)={\left(q_{m,n}^{\mathrm{edge}}\left(t-1\right)\right)}_{n\in N}$: The task queue length of device *m* across all edge nodes; H(t): The load history matrix of edge nodes, used to estimate their future load. The matrix H(t) has dimensions $T^{\mathrm{pre}}\times N$, and the (i,j)-th element of H(t) is defined as:

(12) $${\left\{H\left(t\right)\right\}}_{ij}=B_{j}\left(t-T^{\mathrm{pre}}+i-1\right)$$

Here, $B_{j}\left(t-T^{\mathrm{pre}}+i-1\right)$ represents the number of active service queues at edge node *j* in time slot *t*, reflecting its load intensity. Formally, the state space S can be expressed as the Cartesian product of discrete sets: **$S=\Lambda \times {\left\{0,1,\ldots ,T\right\}}^{2}\times Q^{N}\times {\left\{0,1,\ldots ,M\right\}}^{T^{\mathrm{pre}}\times N}$,** where Λ is the set of possible new task sizes, $Q^{N}$ represents the possible range of queue lengths across *N* edge nodes, and the final term corresponds to the range of values for the elements in the edge load history matrix, where Λ is the set of possible new task sizes; ${\left\{0,1,\ldots ,T\right\}}^{2}$ represents the possible waiting times for computation and transmission queues; $Q^{N}$ represents the possible range of queue lengths across *N* edge nodes; ${\left\{0,1,\ldots ,M\right\}}^{T^{\mathrm{pre}}\times N}$ corresponds to the range of values for the elements in the edge load history matrix. This state definition allows for a complete and precise description of the multidimensional system information required for task offloading decisions in mobile edge computing environments, providing a reliable basis for devising long-term optimal offloading strategies.

### 3.2. Action

At the beginning of time slot *t*, if device *m* receives a new task $k_{m}\left(t\right)$, it must make two decisions: First, determine whether the task should be processed locally or offloaded to an edge node. This decision is represented by the binary variable $x_{m}\left(t\right)\in \left\{0,1\right\}$, where $x_{m}\left(t\right)=1$ indicates that the task enters the local computation queue, and $x_{m}\left(t\right)=0$ indicates that the task enters the transmission queue for offloading to an edge node. Second, if the task is to be offloaded ($x_{m}\left(t\right)=0$), select the target edge node. Let $y_{m}\left(t\right)=\left(y_{m,1}\left(t\right),y_{m,2}\left(t\right),\ldots ,y_{m,n}\left(t\right)\right)$ represent the node selection vector, where $y_{m,n}\left(t\right)\in \left\{0,1\right\}$ is an indicator variable such that $y_{m,n}\left(t\right)=1$ means the task is assigned to edge node *n*, and the constraint $\sum_{n=1}^{N}y_{m,n}\left(t\right)\leq 1$ ensures that the task is offloaded to at most one node or not offloaded at all. Based on these two decisions, the complete action of device *m* at time *t* is defined as

(13) $$a_{m}\left(t\right)=\left(x_{m}\left(t\right),y_{m}\left(t\right)\right)$$

Thus, the joint action space for each terminal is defined as $A={\left\{0,1\right\}}^{1+N}$, indicating that in each time slot, the device can select one optimal action from $2^{1+N}$ possible actions. This action space forms the structural basis for the policy space in subsequent reinforcement learning or optimization problems.

### 3.3. Cost

In a mobile edge computing (MEC) environment, task processing delay and discard penalties together form key performance indicators for system optimization. Specifically, the instantaneous cost of each task consists of two parts: service delay and potential task discard penalties. Let the state of device *m* at time slot *t* be denoted as $s_{m}\left(t\right)$, and the action taken by the device as $a_{m}\left(t\right)$. The task processing delay for task $k_{m}\left(t\right)$ is given by $d_{m}\left(s_{m}\left(t\right),a_{m}\left(t\right)\right)$. The delay is calculated as follows: if the device chooses local processing ($x_{m}\left(t\right)=1$), the task delay is the difference between the local completion time slot and the current time slot:

(14) $$d_{m}\left(s_{m}\left(t\right),a_{m}\left(t\right)\right)=l_{m}^{\mathrm{comp}}\left(t\right)-t+1.$$

If the device chooses offloading to an edge node ($x_{m}\left(t\right)=0$), and the task is offloaded to node *n* ($y_{m,n}\left(t\right)=1$), the task delay is defined as the actual exit delay of the task in the node’s queue:

(15) $$d_{m}\left(s_{m}\left(t\right),a_{m}\left(t\right)\right)=\sum_{n\in N}\sum_{t^{\prime }=t}^{T}I\left(k_{m,n}^{\mathrm{edge}}\left(t^{\prime }\right)=k_{m}\left(t\right)\right)\cdot \left(l_{m}^{\mathrm{edge}}\left(t^{\prime }\right)-t+1\right)$$

Based on the above delay calculation, if the task is successfully processed (either locally or remotely), the instantaneous cost function $c_{m}\left(s_{m}\left(t\right),a_{m}\left(t\right)\right)$ is defined as:

(16) $$c_{m}\left(s_{m}\left(t\right),a_{m}\left(t\right)\right)=d_{m}\left(s_{m}\left(t\right),a_{m}\left(t\right)\right)$$

If the task is discarded due to resource constraints or timeout, a fixed penalty cost C>0 is incurred:

(17) $$c_{m}\left(s_{m}\left(t\right),a_{m}\left(t\right)\right)=C$$

If no new task is generated in the current time slot ($k_{m}\left(t\right)=0$), the instantaneous cost is set to zero ($c_{m}\left(s_{m}\left(t\right),a_{m}\left(t\right)\right)=0$). For convenience, we define the instantaneous cost of device *m* at time *t* as $c_{m}\left(t\right)\equiv c_{m}\left(s_{m}\left(t\right),a_{m}\left(t\right)\right)$.

### 3.4. Problem Model

In mobile edge computing (MEC) systems, the core of task offloading and resource allocation lies in designing a mapping rule from the state space S to the action space A for each terminal device *m*, the strategy $\pi_{m}:S\to A$. After observing the state at time slot *t*, the device selects an action based on $\pi_{m}$, thereby affecting the task delay and drop penalty. To evaluate long-term performance, a discount factor γ∈(0,1] is introduced, and the optimization objective is formulated as minimizing the expected cumulative discounted cost:

(18) $$\pi_{m}^{*}=\arg\min_{\pi_{m}}E\left[\sum_{t\in T}\gamma^{t-1}c_{m}\left(t\right)|\pi_{m}\right]$$

The constraints cover state transitions, queue updates, and cost calculations, as described in Equations (1)–(5), (7)–(10) and (14)–(17). Due to the coupling between edge node load and other devices’ behavior, and the inability to fully access global information, traditional centralized methods struggle with real-time, robust decision-making. To address this, we propose a distributed deep reinforcement learning (DRL) algorithm based on convolutional neural networks (CNN) and the Informer time-series model. This approach leverages CNN to efficiently extract local load fluctuation features and uses Informer to capture long-term temporal dependencies, enhancing the offloading strategy’s awareness of system temporal characteristics and adaptability to heterogeneous tasks, enabling dynamic offloading and scheduling optimization for all terminal devices.

## 4. Deep Reinforcement Learning Task Offloading Algorithm Based on CNN and Informer

This paper proposes a distributed task offloading algorithm based on convolutional neural networks (CNN) and the Informer architecture, utilizing deep reinforcement learning (DRL) to enable mobile devices to autonomously and efficiently make offloading decisions in dynamic environments. Unlike traditional centralized control methods, this approach allows each device to autonomously learn the mapping between environment states and actions, minimizing long-term expected costs under uncertainty. Next, we introduce the neural network architecture for mobile devices, which maps state-action pairs to Q-values. We will then describe the information exchange process between mobile devices and edge nodes.

### 4.1. Network Overview

**Figure 2:**
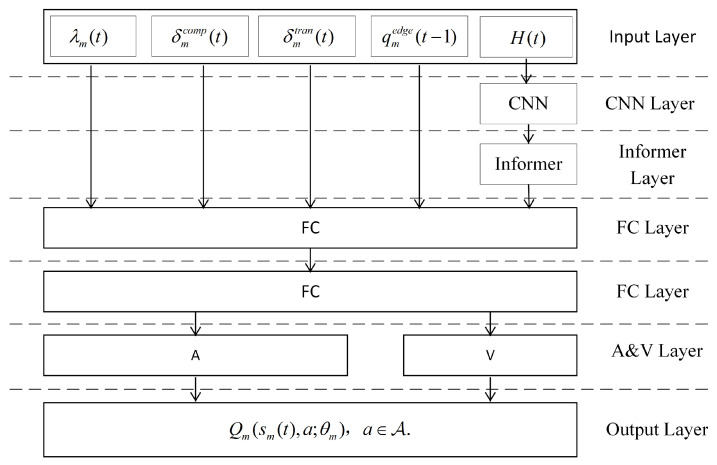
Network architecture of deep reinforcement learning for mobile devices.

The goal of the neural network is to learn a mapping from each state to a set of Q-values, where each Q-value corresponds to an action. For any mobile device m∈M, we consider a seven-layer neural network: an input layer, a CNN layer, an Informer layer, two fully connected (FC) layers, an advantage and value (A&V) layer, and an output layer. The intended network architecture is shown in Figure 2. Let $\theta_{m}$ represent the parameter vector of device *m*’s neural network, which includes the weights for all connections from the input layer to the A&V layer and the biases for all neurons. Specifically, H(t) represents the number of active queues of each edge node in multiple time slots in the past, that is, the number of tasks that are still in a waiting or executing state in that time slot. CNN first convolves H(t) to extract local load fluctuation features, and then inputs these features into the Informer module to model long-term load trends. Finally, the outputs of CNN and Informer are fused with the remaining vectors and input into Dueling DQN to estimate the state-action value function and generate Q-values for each possible action, thereby generating the optimal task offloading decision for each device. The details of each layer are as follows.

In industrial MEC scenarios, the load dynamics of edge nodes usually exhibit multi-scale temporal characteristics, including short-term bursty fluctuations caused by transient task arrivals and long-term periodic or trend-level variations driven by workload patterns and production cycles. To explicitly model these heterogeneous temporal behaviors, we adopt a CNN + Informer hybrid architecture. Specifically, CNN is employed to capture short-term local burst features in the edge load sequences, as convolutional filters are effective in detecting abrupt changes and localized temporal patterns. In contrast, Informer is designed to model long-term dependencies and periodic trends through its self-attention mechanism, which enables efficient global temporal correlation modeling over long sequences. By integrating CNN-based short-term feature extraction with Informer-based long-term trend modeling, the proposed architecture achieves multi-scale temporal representation, thereby improving the robustness and responsiveness of task offloading decisions under highly dynamic edge load conditions.

#### 4.1.1. Multi-Scale Temporal Modeling Motivation

In industrial MEC environments, edge node load dynamics often exhibit multi-scale temporal characteristics. Short-term load bursts may arise from transient task arrivals or sudden workload spikes, while long-term load trends are influenced by production cycles and periodic system operations.

To effectively capture these heterogeneous temporal patterns, we adopt a hybrid CNN–Informer architecture. CNN is well suited for extracting local temporal patterns from short historical windows due to its ability to detect localized fluctuations and bursty behaviors. In contrast, Informer leverages the self-attention mechanism and ProbSparse attention to efficiently model long-range temporal dependencies and global load trends.

By integrating CNN-based short-term feature extraction with Informer-based long-term temporal modeling, the proposed architecture enables multi-scale perception of edge load dynamics, providing richer state representations for reinforcement learning-based task offloading decisions.

#### 4.1.2. Input Layer

This layer is responsible for receiving the device’s state information and performing initial preprocessing for subsequent network modules. We feed the state vector $s_{m}\left(t\right)$, consisting of $\lambda_{m}\left(t\right)$, $\delta_{m}^{\mathrm{comp}}\left(t\right)$, $\delta_{m}^{\mathrm{tran}}\left(t\right)$, and $q_{m}^{\mathrm{edge}}\left(t-1\right)$, into the fully connected layer. The local load features are derived from the load history matrix H(t) of edge nodes, whose elements represent the number of active task queues of each edge node within a continuous time slice, reflecting the short-term load fluctuations of edge nodes. The load history matrix H(t) is input into the CNN layer. This approach integrates both the static queue information at the current time and the temporal features of historical load, providing the deep reinforcement learning (DRL) agent with a more comprehensive and refined environmental representation.

#### 4.1.3. CNN Layer

The CNN layer aims to extract local temporal features from the load history matrix H(t) of edge nodes to capture short-term load fluctuation patterns. Specifically, the historical load sequence ${\left\{H_{i,n}\left(t\right)\right\}}_{i=1}^{T^{\mathrm{pre}}}$ of the *n*-th node is used as input to a 1D convolutional network. The convolution kernel size is 3, the number of filters is 32, the stride is 1, and the activation function is ReLU, producing the output feature matrix:

(19) $$C_{n}\left(t\right)=\mathrm{CNN}\left({\left\{H_{i,n}\left(t\right)\right\}}_{i=1}^{T^{\mathrm{pre}}}\right)\in R^{T_{c}\times d_{c}}$$

Unlike the raw observations, $C_{n}\left(t\right)$ represents the locally abstracted feature pattern after convolution, effectively enhancing the perception of short-term load variations in subsequent modules.

#### 4.1.4. Informer Layer

Compared with conventional recurrent time-series models such as LSTM and GRU, Informer exhibits several structural advantages for modeling edge load dynamics in industrial MEC environments. First, LSTM/GRU process sequences in a step-by-step recurrent manner, which may suffer from limited parallelization and higher computational cost when modeling longer historical load windows. In contrast, Informer leverages self-attention mechanisms that enable parallel computation and more efficient long-sequence modeling. Second, Informer adopts the ProbSparse attention mechanism, which selectively focuses on the most informative query–key pairs, significantly reducing computational complexity from quadratic to near-linear with respect to sequence length. This property is particularly suitable for real-time industrial scenarios where long-term load trends must be captured under strict latency constraints. Finally, unlike recurrent models that may gradually lose sensitivity to long-range dependencies, Informer explicitly models global temporal correlations through attention, improving robustness to sudden and non-stationary load fluctuations. These structural characteristics make Informer a more appropriate choice for multi-scale load perception in dynamic MEC environments.

**Figure 3:**
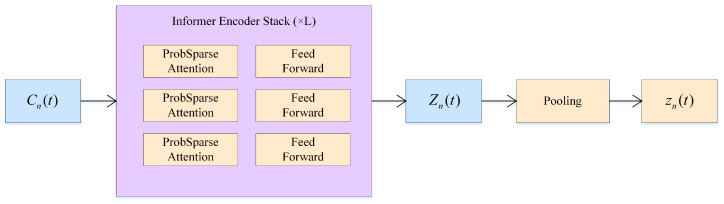
Informer processing flowchart.

This layer models the long-term temporal dependencies in the load state of edge nodes. After obtaining the local temporal features $C_{n}\left(t\right)$ for each node from the CNN layer, the Informer encoder is used to model the long-term dependencies, capturing load trends and global structural features. Informer employs the ProbSparse self-attention mechanism, balancing the efficiency and expressiveness of long-sequence modeling. The process is illustrated in Figure 3.

Specifically, in the Informer module, the sequence length is set to 20, the number of attention heads to 4, and the hidden dimension to 64. The matrix $C_{n}\left(t\right)$ is input into a stack of causal self-attention layers and a feed-forward network, producing the output sequence:

(20) $$Z_{n}\left(t\right)=\mathrm{Informer}\left(C_{n}\left(t\right)\right)\in R^{T_{x}\times d_{z}}$$

By taking the output at the final time step or applying average pooling, we obtain the node-level global feature vector:

(21) $$z_{n}\left(t\right)=\mathrm{Pooling}\left(Z_{n}\left(t\right)\right)\in R^{d_{z}}$$

The vector $z_{n}\left(t\right)$ is then concatenated with the local state variables, forming a part of the final state vector:

(22) $${\tilde{s}}_{m}\left(t\right)=\left(\lambda_{m}\left(t\right),\delta_{m}^{\mathrm{comp}}\left(t\right),\delta_{m}^{\mathrm{tran}}\left(t\right),q_{m}^{\mathrm{edge}}\left(t-1\right),{\left\{z_{n}\left(t\right)\right\}}_{n\in N}\right)$$

#### 4.1.5. FC Layer

After feature extraction by the CNN and Informer modules, the network includes two fully connected (FC) layers to map the fused state information to action value estimates. The first FC layer receives all state variables except for the load history matrix H(t) and the edge node load matrix ${\left\{z_{n}\left(t\right)\right\}}_{n\in N}$ extracted by the CNN + Informer module. This layer performs initial fusion of state information from different sources and maps it to a latent space. The second FC layer further abstracts high-level features from the first layer’s output and connects them to all neurons in the Advantage & Value (A&V) module. This module, based on the Dueling DQN structure, separately estimates the state value function V(s) and the action advantage function A(s,a), which are combined to provide the final Q-value estimate for the action. This hierarchical FC structure ensures sufficient feature abstraction from the raw state to the Q-value representation, while enhancing the stability and generalization of policy learning. Finally, the second FC layer outputs a latent vector representation, which is forwarded to the dueling A&V heads introduced in the next subsection.

#### 4.1.6. Advantages & Value Layer and Output Layer

Given the latent representation produced by the FC stack, the A&V module implements the dueling architecture: V(s) estimates the state value and A(s,a) captures the relative benefit of action *a*; they are combined to yield Q(s,a). The A&V layer consists of two parallel subnetworks: Network A (for learning action advantages) and Network V (for learning state values). Both the A&V layer and the output layer use the Dueling-DQN architecture to compute the Q-values for each action. This structure decomposes the Q-value into two components: the state value, which reflects the long-term value of the current state, and the action advantage, which captures the incremental benefit of taking a specific action in that state. By estimating these components separately and combining them, the final Q-value is obtained, improving the convergence efficiency and stability of the policy. For each mobile device m∈M, we define an advantage network $A_{m}\left(s_{m}\left(t\right),a;\theta_{m}\right)$ and a state-value network $V_{m}\left(s_{m}\left(t\right);\theta_{m}\right)$ which respectively estimate the action-advantage for taking action a∈A and the value of state $s_{m}\left(t\right)$. Both networks share the parameter vector $\theta_{m}$, which is learned via a deep reinforcement learning algorithm. Under the Dueling-DQN architecture, the Q-value for device *m* at state $s_{m}\left(t\right)$ and action *a* is given by:

(23) $$Q_{m}\left(s_{m}\left(t\right),a;\theta_{m}\right)=V_{m}\left(s_{m}\left(t\right);\theta_{m}\right)+\left(A_{m}\left(s_{m}\left(t\right),a;\theta_{m}\right)-\frac{1}{\left|A\right|}\sum_{a^{\prime }\in A}A_{m}\left(s_{m}\left(t\right),a^{\prime };\theta_{m}\right)\right)$$

In other words, the Q-value is computed as the state value plus the action’s advantage minus the mean advantage over all actions. The neural network of device *m*, parameterized by $\theta_{m}$, thus implements a function so that for any observed state $s_{m}\left(t\right)$ and action a∈A, it outputs the long-term expected return $Q_{m}\left(s_{m}\left(t\right),a;\theta_{m}\right)$.

### 4.2. Optimization Algorithm Based on Deep Reinforcement Learning

To offload computation from terminal devices, we adopt a distributed DRL framework in which edge nodes assist model training. Each device m∈M selects the edge node $n_{m}$ offering the best channel and uploads its experience tuple $\left(s_{m}\left(t\right),a_{m}\left(t\right),c_{m}\left(t\right),s_{m}\left(t+1\right)\right)$ for time slot *t* over a high-speed link. At each edge node *n*, for each associated terminal device set $M_{n}$, two sets of deep Q-networks with the same network structure but independent parameters are maintained: the *evaluation network *${\mathrm{Eval}\_\mathrm{Net}}_{m}$ (parameters $\theta_{m}^{\mathrm{Eval}}$) is used to calculate the Q-values under the current policy in real time to guide action selection; and the *target network *${\mathrm{Target}\_\mathrm{Net}}_{m}$ (parameters $\theta_{m}^{\mathrm{Target}}$) is used to generate stable training targets. Each node also keeps an experience replay buffer $D_{m}$. Periodically, a mini-batch is sampled from $D_{m}$, and $\theta_{m}^{\mathrm{Eval}}$ is updated by minimizing the squared error between ${\mathrm{Eval}\_\mathrm{Net}}_{m}$ outputs and the targets from ${\mathrm{Target}\_\mathrm{Net}}_{m}$. Every *K* steps, $\theta_{m}^{\mathrm{Target}}$ is synchronized to $\theta_{m}^{\mathrm{Eval}}$. This design lets terminals make lightweight, real-time action decisions locally, while heavyweight gradient computations and parameter updates occur at edge nodes—thereby significantly lowering terminal compute and energy demands and leveraging edge resources to accelerate and stabilize policy learning.

#### 4.2.1. Algorithms Used in Mobile Devices

This study uses a multi-episode training method, where #Episodes represents the total number of episodes. At the beginning of each round, mobile device m∈M is initialized with the following state: ${\tilde{s}}_{m}\left(1\right)=\left(\lambda_{m}\left(1\right),\delta_{m}^{\mathrm{comp}}\left(1\right),\delta_{m}^{\mathrm{train}}\left(1\right),q_{m}^{\mathrm{edge}}\left(0\right),z_{n}\left(1\right)\right)$ where $q_{m}^{\mathrm{edge}}\left(0\right)$ applies for all n∈N and $z_{n}\left(1\right)$ is a scheduling-aware vector capturing task history and resource state. Each episode comprises the time-slot set T. At the beginning of slot *t*, if device *m* receives a new task $k_{m}\left(t\right)$, it requests parameters from its selected edge node $n_{m}$. The edge node evaluates its Q-network ${\mathrm{Eval}\_\mathrm{Net}}_{m}$ (parameters $\theta_{m}^{\mathrm{Eval}}$) and returns the Q-values. Device *m* then selects action $a_{m}\left(t\right)$, specifically, as follows:

(24) $$a_{m}\left(t\right)=\left\{\begin{array}{c} \mathrm{Randomly}\;\mathrm{select}\;\mathrm{an}\;\mathrm{action}\;\mathrm{from}\;A\;\mathrm{with}\;\mathrm{probability}\;\epsilon \\ \arg\min_{a\in A}Q_{m}^{\mathrm{Eval}}\left(s_{m}\left(t\right),a;\theta_{m}^{\mathrm{Eval}}\right)\;\mathrm{with}\;\mathrm{probability}\;1-\epsilon \end{array}\right.$$

Let $Q_{m}^{\mathrm{Eval}}\left(s_{m}\left(t\right),a;\theta_{m}^{\mathrm{Eval}}\right)$ denote the evaluation network’s estimate of the Q-value for state-action pair $\left(s_{m}\left(t\right),a\right)$ under parameters $\theta_{m}^{\mathrm{Eval}}$. We employ an ϵ-greedy policy: with probability ϵ, the device explores by selecting a random action; otherwise, with probability 1−ϵ, it exploits by choosing $a_{m}\left(t\right)=\arg\min_{a\in A}Q_{m}^{\mathrm{Eval}}\left(s_{m}\left(t\right),a;\theta_{m}^{\mathrm{Eval}}\right)$. Upon entering time slot t+1, device *m* observes the new state $s_{m}\left(t+1\right)$. Since task execution and transmission may span multiple time slots, the instantaneous cost $c_{m}\left(t\right)$ may not be immediately available at time *t*. Therefore, the cost associated with a task can only be determined when the task is completed or discarded due to timeout. To improve clarity and avoid symbolic ambiguity, we explicitly distinguish the task arrival time from the current decision time. Let τ denote the arrival time slot of a task generated by device *m*, and let *t* denote the current time slot. We define an indicator function to represent whether a task has been completed or discarded by a given time slot:

(25) $$I_{\mathrm{done}}\left(m,\tau ,t\right)=\left\{\begin{array}{cc} 1, & \mathrm{if}\;\mathrm{task}\;k_{m}\left(\tau \right)\;\mathrm{is}\;\mathrm{completed}\;\mathrm{or}\;\mathrm{discarded}\;\mathrm{by}\;\mathrm{time}\;\mathrm{slot}\;t,\\ 0, & \mathrm{otherwise}. \end{array}\right.$$

Based on the above definition, we denote by ${\tilde{T}}_{m,t}$ the set of task arrival time slots whose corresponding tasks have been completed or discarded by the end of time slot *t*, defined as:

(26) $${\tilde{T}}_{m,t}=\left\{\tau \;|\;1\leq \tau \leq t,\;\lambda_{m}\left(\tau \right)>0,\;I_{\mathrm{done}}\left(m,\tau ,t\right)=1\right\}.$$

For each $\tau \in {\tilde{T}}_{m,t}$, device *m* constructs an experience tuple $(s_{m}\left(\tau \right),a_{m}\left(\tau \right),c_{m}\left(\tau \right),s_{m}\left(\tau +1\right)$ and uploads it to the corresponding edge node for experience replay and network training. At the start of slot t+1, device *m* retrieves the cost set $\left\{c_{m}\left(\tau \right)|\tau \in {\tilde{T}}_{m,t}\right\}$. Then, for each $\tau \in {\tilde{T}}_{m,t}$, device *m* constructs the experience tuple $(s_{m}\left(\tau \right),a_{m}\left(\tau \right),c_{m}\left(\tau \right),s_{m}\left(\tau +1\right)$ and uploads it to edge node $n_{m}$ for parameter updates and storage in the replay buffer. This division of labor—local decision-making and data upload by the terminal, with model training and gradient computation performed at the edge—substantially reduces the devices’ computational burden and accelerates training. The pseudocode flowchart is shown in Algorithm 1.
**Algorithm 1** CNN + Informer + D3QN Algorithm at Device m∈M  1:**for** episode from 1 to #Episodes **do**  2:   Initialize $s_{m}\left(1\right)$;  3:   **for** time slot t∈T **do**  4:     **if** device *m* has a new task arrival $k_{m}\left(t\right)$ **then**  5:        Send a parameter request to edge node $n_{m}$; Receive network parameter vector $\theta_{m}^{\mathrm{Eval}}$. Select an action $a_{m}\left(t\right)$ according to (24);  6:     **end if**  7:     Observe the next state $s_{m}\left(t+1\right)$ and a set of costs $\left\{c_{m}\left(\tau \right)|\tau \in {\tilde{T}}_{m,t}\right\}$;  8:     **for** each task $k_{m}\left(\tau \right)$ with $\tau \in {\tilde{T}}_{m,t}$ **do**  9:        Send $\left(s_{m}\left(\tau \right),a_{m}\left(\tau \right),c_{m}\left(\tau \right),s_{m}\left(\tau +1\right)\right)$ to $n_{m}$10:     **end for**11:   **end for**12:**end for**

#### 4.2.2. Algorithms Used by Edge Nodes

For each mobile device *m* in the device set $M_{n}$, initialize the experience replay pool $D_{m}$ and the corresponding evaluation network (Eval_Net_*m*_) and target network (Target_Net_*m*_). Edge node *n* performs the following operations: When the edge node receives a parameter request (parameter_request) from mobile device *m*, the node sends the current parameters $\theta_{m}^{\mathrm{Eval}}$ of its evaluation network back to the device. When it receives the experience sample quadruple $\left(s_{m}\left(t\right),a_{m}\left(t\right),c_{m}\left(t\right),s_{m}\left(t+1\right)\right)$ from the device, it stores them in the replay pool $D_{m}$ on a first-in, first-out (FIFO) basis and limits the pool capacity to control storage overhead. Specifically, a batch of experience samples (denoted as set *J*) is randomly sampled from cache $D_{m}$, and parameter updates are performed with the objective of minimizing the error between the Q-values predicted by the evaluation network and the target Q-values provided by the target network. The loss function is defined as follows:

(27) $$L\left(\theta_{m}^{\mathrm{Eval}},{\tilde{Q}}_{m}^{\mathrm{Target}}\right)=\frac{1}{\left|J\right|}\sum_{i\in J}{\left(Q_{m}^{\mathrm{Eval}}\left(s_{m}\left(i\right),a_{m}\left(i\right);\theta_{m}^{\mathrm{Eval}}\right)-{\tilde{Q}}_{m,i}^{\mathrm{Target}}\right)}^{2}$$

Among them, |J| is the sample set size. Gradient descent optimization is performed on the loss function using the backpropagation algorithm to complete the evaluation of network parameter updates. The target Q value, ${\tilde{Q}}_{m,i}^{\mathrm{Target}}$, is calculated using Double DQN technology to improve the accuracy of long-term cost estimates, defined as follows:

(28) $${\tilde{Q}}_{m,i}^{\mathrm{Target}}=c_{m}\left(i\right)+\gamma Q_{m}^{\mathrm{Target}}\left(s_{m}\left(i+1\right),a_{i}^{\mathrm{Next}},\theta_{m}^{\mathrm{Target}}\right)$$

Among them:

(29) $$a_{i}^{\mathrm{Next}}=\underset{a\in A}{\mathrm{argmin}}Q_{m}^{\mathrm{Eval}}\left(s_{m}\left(i+1\right),a;\theta_{m}^{\mathrm{Eval}}\right)$$

To avoid the overestimation bias inherent in standard DQN, we adopt the Double DQN mechanism when computing the target Q-value. Specifically, the evaluation network is used to select the next action $a_{i}^{\mathrm{Next}}=\arg\min_{a\in A}Q_{m}^{\mathrm{Eval}}\;\left(s_{m}\left(i+1\right),a;\theta_{m}^{\mathrm{Eval}}\right)$, while the target network is employed to evaluate the corresponding Q-value. This decoupled action selection and evaluation strategy effectively mitigates the overestimation problem caused by max-operator bias in traditional DQN, leading to more stable and accurate value estimation during training.

Under the next state $s_{m}\left(i+1\right)$, the optimal action predicted by the evaluation network is assessed. To ensure training stability, the evaluation network parameters $\theta_{m}^{\mathrm{Eval}}$ are soft-updated to the target network $\theta_{m}^{\mathrm{Target}}$ at fixed intervals to reduce valuation oscillations during training. Through the above mechanism, edge nodes can utilize centrally stored historical experience to continuously optimize the Q-value prediction accuracy of the evaluation network, while terminal devices only need to periodically retrieve the latest parameters and perform lightweight action selection. This achieves effective separation and collaborative optimization of computational load while ensuring training stability and accuracy. The pseudocode flowchart is shown in Algorithm 2.
**Algorithm 2** CNN + Informer + D3QN Algorithm at Edge n∈N  1:Initialize replay memory $D_{m}$ for each device $m\in M_{n}$ and set Count:=0;  2:Initialize ${\mathrm{Eval}\_\mathrm{Net}}_{m}$ and ${\mathrm{Target}\_\mathrm{Net}}_{m}$ with random parameters $\theta_{m}^{\mathrm{Eval}},\theta_{m}^{\mathrm{Target}}$;  3:**while** true **do**  4:   **if** receive a parameter request from device $m\in M_{n}$ **then**  5:     send $\theta_{m}^{\mathrm{Eval}}$ to device *m*  6:   **end if**  7:   **if** receive an experience $\left(s_{m}\left(t\right),a_{m}\left(t\right),c_{m}\left(t\right),s_{m}\left(t+1\right)\right)$ from device $m\in M_{n}$ **then**  8:     Store $\left(s_{m}\left(t\right),a_{m}\left(t\right),c_{m}\left(t\right),s_{m}\left(t+1\right)\right)$ in $D_{m}$; Sample a set of experiences (denoted by *J*) from $D_{m}$;  9:     **for** each experience i∈J **do**10:        Obtain experience $\left(s_{m}\left(i\right),a_{m}\left(i\right),c_{m}\left(i\right),s_{m}\left(i+1\right)\right)$; Compute ${\tilde{Q}}_{m,i}^{\mathrm{Target}}$ according to (27);11:     **end for**12:     Set vector ${\tilde{Q}}_{m}^{\mathrm{Target}}:=\left({\tilde{Q}}_{m,i}^{\mathrm{Target}},i\in J\right)$; Update $\theta_{m}^{\mathrm{Eval}}$ to minimize $L(\theta_{m}^{\mathrm{Eval}},{\tilde{Q}}_{m}^{\mathrm{Target}})$ in (26);13:     Count:=Count+1;14:     **if** mod(Count,Replace_Threshold)=0 **then**15:        $\theta_{m}^{\mathrm{Target}}:=\theta_{m}^{\mathrm{Eval}}$16:     **end if**17:   **end if**18:**end while**

We adopt a decision-making RPC and empirical pipeline: when tasks start to arrive in a slot, the terminal reports the state $s_{m}\left(t\right)$, and the edge returns $Q_{m}\left(s_{m}\left(t\right),a;\theta_{m}\right)$; after tasks are completed or timed out, the quadruples are uploaded to the replay pool, training is completed at the edge, and the terminal does not issue weights. Assuming a slot duration of Δ=0.1s, task arrival probability: 0.3, number of edge nodes *N*: 5 units, a history window of L=20, and a state dimension of $d_{s}=108$. Communication (per device): The decision-making RPC is approximately 456B/time (uplink 432B status + downlink 24B Q); the experience upload is approximately 870B/item. The triggering frequency is about 3 times/s, and the experience is about 2.9 items/s; the total is approximately 3.9KB/s (≈31.2 kbps). The total control plane for 50 devices is ≈195 KB/s (≈1.56 Mbps). Update frequency: batch=32, training on arrival; target network synchronization every K=200 steps.

## 5. Experimental Evaluation

This section validates the effectiveness of the proposed CNN + Informer-based deep reinforcement learning (DRL) offloading strategy in an industrial environment through simulation experiments. Four representative control algorithms are selected for comparison: the selected algorithms include: No Offloading (No.OffL), No OffL serves as a lower-bound reference, where all tasks are executed locally. Random Offloading (R. OffL), R. OffL represents a non-intelligent strategy, providing a comparison against our learning-based method. Potential-Game Optimal Response Algorithm (PGOA) is a best-response potential-game heuristic that monotonically increases a global potential and typically converges, and Utility- and Load-Oriented Offloading (ULOOF) is a capacity-based heuristic that selects the node with the highest utility computed from residual CPU, queue length, and uplink rate while avoiding overload. Among them, the PGOA population size is 50, and the objective function weight of ULOOF is set to (0.5, 0.5). Delay-sensitive tasks are computing tasks with strict real-time constraints in the industrial Internet of Things, and thus their performance evaluation focuses on both task completion and service timeliness. To comprehensively evaluate the performance of each method, we use the following two key performance metrics: Task Drop Rate: the ratio of the number of dropped tasks to the total number of tasks received; Average Processing Latency: the average processing delay of all completed tasks, measuring the system’s service efficiency.

The simulation parameters are set as follows: Number of mobile devices *M*: 50 units, number of edge nodes *N*: 5 units, time slot length Δ: 0.1 s, local computing power of each device $f_{m}^{\mathrm{device}}$: 2.5 GHz (seconds), processing capacity of each edge node $f_{n}^{\mathrm{edge}}$: 41.8 GHz (seconds), wireless transmission rate from each device to each edge node $f_{m,n}^{\mathrm{tran}}$: 14 Mbps, Task size $\lambda_{m}\left(t\right)$: takes discrete values in the range {2.0, 2.1, …, 5.0} Mbits, processing density $\rho_{m}$: 0.297 GFLOP/bit, task deadline delay $\tau_{m}$: 10 time slots (i.e., 1 s), task arrival probability: 0.3.

In industrial production processes, network bandwidth, node load, and task deadline constraints are subject to stricter requirements. Therefore, this simulation specifically adjusts these parameters to simulate the edge computing pressure and dynamics in real industrial scenarios. Through the above comparisons, we aim to validate the robustness and performance improvement of the proposed algorithm in complex industrial environments.

The parameter settings for DQN are as follows: replay buffer size: 500; batch size: 32; Optimizer: Root Mean Square Propagation; learning rate: 0.01; γ: 0.9; Target-network update period: 200; ϵ-greedy schedule: 0.99; training horizon: 110, number of seeds: 42.

To quantify the actual impact of the communication-computation tradeoff on the system, we recorded the running time and memory occupation during the steady-state phase of inference and training. Memory at the edge (single node): Playback pool ≈ 4.2 MB; Model + Optimizer ≈ 18–20 MB; Temporary activation ≈ 15 MB; Total ≈ 40 MB. Running time: Inference Qs ≈ 0.18 ms/time; Training (batch = 32) ≈ 3.5 ms/step. Terminal: No training; Inference only takes argmax <0.05 ms/step, memory <1 MB.

In all the tables that appear below: The C-o represents the CNN-only; The I-o represents the Informer-only; The w /o-d represents the w/o dueling head; The C-I-D3QN represents the CNN-Informer-D3QN.

### 5.1. Algorithm Convergence and Performance

**Figure 4:**
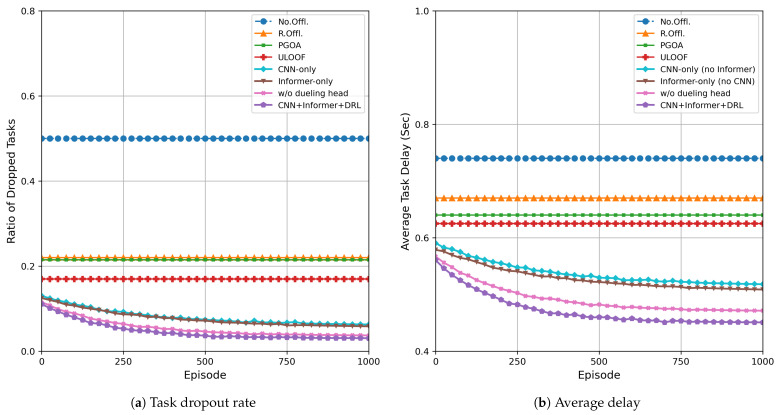
Performance evaluation of multiple training episodes: (**a**) task dropout rate; (**b**) average delay.

Figure 4 shows a comparison of the performance of the proposed deep reinforcement learning offloading algorithm based on CNN + Informer and four benchmark methods during multi-round training. Figure 4a shows the trend of task discard rates for each algorithm as the number of rounds increases; Figure 4b shows the evolution of the average task processing delay for each algorithm as the number of rounds increases. The specific values are shown in Table 2 and Table 3. As can be seen from the figure, this method begins to stabilize after approximately 350 training iterations, while other benchmark algorithms remain nearly unchanged throughout the process. In terms of task dropout rate (Figure 4a): this method eventually stabilizes at approximately 0.03, representing an improvement of approximately 82.3% to 94% compared to No Offl., R. Offl., PGOA, and ULOOF, significantly reducing the risk of task dropout. In terms of average latency (Figure 4b): after convergence, the average latency of this method is approximately 0.45 s, reducing the average latency by 28% to 39.2% compared to the baseline methods, indicating its superior robustness and efficiency under resource-constrained and load-fluctuating conditions. To enhance reproducibility and statistical credibility, the steady-state results at the final training episode are averaged over five independent runs and reported as mean ± standard deviation, as summarized in Table 4.

In summary, the experimental results fully validate the performance advantages of the proposed algorithm in multi-device collaborative offloading scenarios in industrial environments, particularly in terms of reducing task discard rates and shortening processing delays. We use the Jain Fairness Index to evaluate the fairness between devices, and the Jain Fairness Index formula is as follows:

(30) $$J=\frac{{\left(\sum_{i=1}^{n}x_{i}\right)}^{2}}{n\cdot \sum_{i=1}^{n}x_{i}^{2}}$$

After calculation, the Jain fairness index is about 0.92, which is close to 1, indicating that the distribution of task retention rates among different devices is relatively balanced, and the system improves overall performance without sacrificing device fairness.

### 5.2. Task Arrival Probability Performance

**Figure 5:**
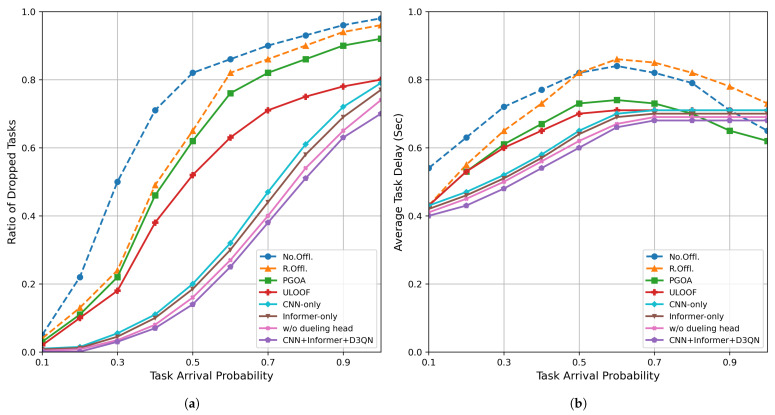
Performance evaluation under different task arrival probabilities: (**a**) task dropout rate; (**b**) average delay.

Figure 5 shows the performance changes of each strategy under different task arrival probabilities, where Figure 5a shows the change in task discard rate with arrival probability, and Figure 5b shows the change in average processing delay with arrival probability. The specific values are shown in Table 5 and Table 6.

As shown in Figure 5a, the task dropout rates of each method vary with the task arrival probability. When the arrival probability increases from 0.1 to 0.5, the baseline method’s drop rate surges from nearly 0 to over 50%, while our algorithm consistently maintains the drop rate below 20%. In the moderate load range (0.3–0.8), our algorithm reduces the drop rate by approximately 39.2% on average compared to the baseline method, demonstrating stronger robustness and scheduling capabilities.

As shown in Figure 5b, the average delay of each method changes under different task arrival probabilities. When the arrival probability increases from 0.1 to 0.4, the average delay of our algorithm increases by only approximately 25.9%, while the control algorithm increases by at least 33.9%. When the arrival rate further increases to 0.6 or higher, some benchmark methods exhibit a decrease in delay. This is because, after a large number of tasks are discarded, only the delays of the few completed tasks are counted. Although the delay of our algorithm is slightly higher than some benchmarks at this point, its lower discard rate ensures a higher task retention rate and fairness, resulting in overall performance that still outperforms the control scheme. The above results indicate that when system load increases, the proposed deep reinforcement learning offloading strategy based on CNN + Informer can more stably control task discard and exhibit better smoothness in latency growth, thereby validating its generalizability and reliability in industrial environments.

### 5.3. Mobile Device Quantity Analysis

**Figure 6:**
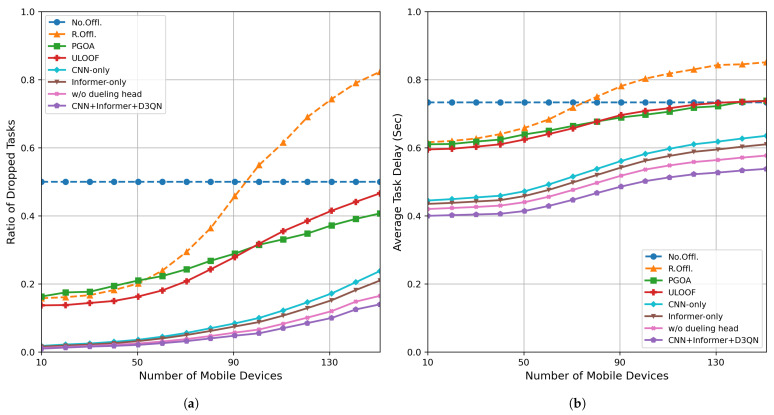
Performance evaluation under different numbers of mobile devices: (**a**) task dropout rate; (**b**) average delay.

Figure 6 shows the task drop rate and average delay for each strategy under different numbers of mobile devices. Figure 6a shows the trend of task drop rate with device scale, and Figure 6b shows the change in average processing delay with device scale.

As shown in Figure 6a, the algorithm consistently maintains the drop rate at a low level as the number of mobile devices increases, achieving an average reduction of approximately (69.9∼82.9%) compared to the Random Offloading (R. Offl.), PGOA, and ULOOF methods. This is primarily attributed to the CNN + Informer model’s precise modeling and dynamic adaptation of edge node loads, enabling timely detection and mitigation of system bottlenecks. Figure 6b further reveals the impact of increasing mobile device counts on system average processing latency. Under the No Offl. strategy, latency remains largely unchanged; however, for other offloading methods, as device concurrency increases, edge node congestion leads to a continuous rise in average latency. Our method reduces average latency by approximately 27% compared to the PGOA and ULOOF methods by more evenly distributing computational tasks, demonstrating better scheduling stability and congestion resistance. In summary, in high-concurrency industrial IoT scenarios, the proposed CNN-Informer-based deep reinforcement learning offloading strategy effectively utilizes system redundant computational resources, significantly improving system service quality and task retention capability.

### 5.4. Impact of Mobile Device Computing Power

**Figure 7:**
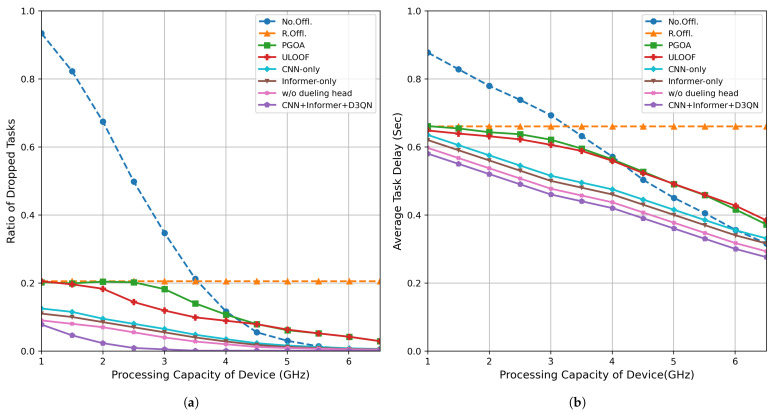
Performance evaluation of mobile devices with different computing capabilities: (**a**) task dropout rate; (**b**) average delay.

Figure 7 shows the performance comparison of various offloading strategies under different local computing capabilities of terminals, where Figure 7a shows the task discard rate as a function of local processing frequency, and Figure 7b shows the average task processing latency as a function of local processing frequency. The specific values are shown in Table 7.

Figure 7a shows that as the local processing frequency increases from 1.0 GHz to 3.5 GHz, the dropout rates of all methods decrease, but the decrease in our method is the most significant. When the frequency reaches 3.5 GHz, the dropout rate of our method drops to 0.005, representing a reduction of approximately 95% compared to PGOA and ULOOF, demonstrating its efficient utilization and scheduling optimization capabilities of local resources in high-performance terminal scenarios.

Figure 7b further analyzes the performance of each method in terms of average task processing latency under different processing capabilities. As local computing power increases, average processing latency generally decreases; at 3.5 GHz, the average latency of our method is approximately 26% lower than that of PGOA and ULOOF, respectively, indicating that it can more fully utilize local computing resources and reduce the additional latency caused by edge offloading.

In summary, the deep reinforcement learning offloading strategy based on CNN+ Informer proposed in this study can further enhance task retention rates and reduce processing delays under conditions of improved terminal computing capabilities, demonstrating excellent adaptability and scheduling efficiency.

### 5.5. Impact of Edge Node Computing Power

**Figure 8:**
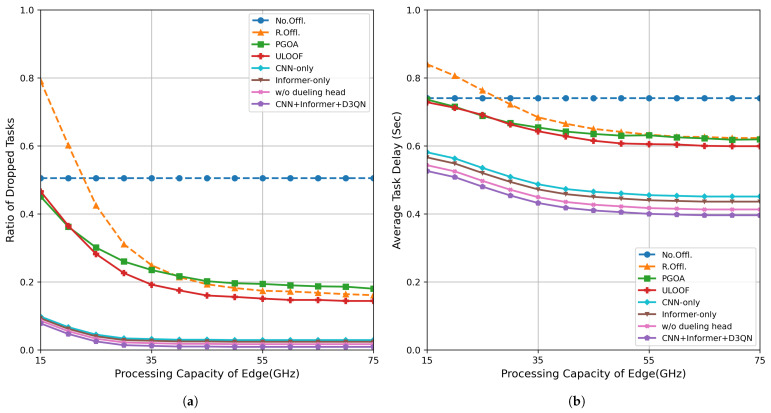
Performance evaluation under different computing capacities of edge nodes: (**a**) task dropout rate; (**b**) average delay.

The computing power of edge nodes plays a decisive role in the service performance of offloading tasks, especially in scenarios with high task density and intense resource competition, where its impact is particularly significant. Figure 8 shows the trends in task drop rates and average processing delays for different offloading methods under different edge node processing frequency configurations. The specific values are shown in Table 8.

As shown in the figure, except for the “No Offloading” strategy, all offloading methods exhibit a decreasing trend in both task drop rate and average latency as the edge node frequency increases from low to high, indicating that more tasks are successfully offloaded for processing, thereby alleviating the computational load on the terminal.

Figure 8a shows that when the edge node frequency reaches 15 GHz, the task drop rate of our method is reduced by an average of 82.7–90.1% compared to other benchmark algorithms, significantly improving the system’s reliability and throughput. Figure 8b illustrates that under the same configuration, the average processing latency of our method is reduced by 27.7–37.3% compared to the benchmark algorithms, further validating its optimization effects on task scheduling and resource allocation in high-concurrency scenarios. In summary, the experimental results demonstrate that the proposed CNN + Informer-based deep reinforcement learning offloading strategy consistently performs well across different edge node computational capabilities, particularly exhibiting outstanding task retention and latency control capabilities in resource-constrained or high-load environments.

From a practical deployment perspective, it is important to examine the computational overhead introduced by the CNN+Informer+D3QN architecture. In the proposed framework, model inference and training are performed at edge nodes, while terminal devices only execute lightweight action selection, which significantly reduces on-device computational burden. In terms of inference latency, the forward pass of the CNN–Informer–D3QN network can be completed within the millisecond level on typical edge computing hardware, which is well aligned with the time-slot duration considered in industrial MEC systems. Moreover, the Informer architecture employs the ProbSparse attention mechanism, effectively reducing the computational complexity of long-sequence modeling and mitigating the overhead compared to conventional full self-attention mechanisms. In terms of inference latency, the forward pass of the CNN–Informer–D3QN network takes approximately 0.18 ms per decision on typical edge hardware, while training with batch size 32 requires about 3.5 ms per update, which is well within the 0.1 s slot duration considered in industrial MEC systems. The memory footprint at each edge node is approximately 40 MB, while terminal devices require less than 1 MB, ensuring lightweight deployment. Although the proposed model introduces additional complexity compared to heuristic baselines, the resulting computational overhead remains acceptable for edge nodes and does not hinder real-time task offloading decisions. Therefore, the proposed approach achieves a favorable trade-off between decision accuracy and practical deployability in industrial MEC environments.

This chapter systematically evaluates the proposed CNN+Informer-driven deep reinforcement learning offloading strategy through extensive simulation experiments. The evaluation covers multiple dimensions, including task arrival probability, device scale, and computational capabilities of both local devices and edge nodes, with comprehensive comparisons against baseline methods. The experimental results demonstrate that under conditions of high task arrival rates and large-scale concurrent terminals, the proposed strategy consistently maintains the lowest task drop rate, significantly mitigating the risk of task loss compared to traditional approaches. Under medium-to-high load conditions, the system’s end-to-end latency is substantially optimized, highlighting the method’s effectiveness in supporting latency-sensitive applications. Furthermore, the strategy demonstrates the capability to dynamically identify and leverage available computational resources when the processing power of either local devices or edge nodes is enhanced, achieving optimal task scheduling outcomes. In comparison to alternative methods, it exhibits superior adaptability and stability within heterogeneous computational environments, along with enhanced task retention capacity and improved response timeliness. In summary, the proposed distributed deep reinforcement learning offloading strategy demonstrates exceptional robustness and adaptability across various typical scenarios. It effectively enhances both task retention rates and latency performance in MEC systems within industrial IoT contexts, thereby establishing a solid foundation for real-time and reliable edge computing scheduling.

## 6. Conclusions

This paper addresses the task offloading challenge in cloud-edge-end collaborative systems within smart manufacturing environments, proposing a distributed offloading algorithm that integrates convolutional neural networks (CNN) with the Informer architecture using deep reinforcement learning (DRL). The decentralized design enables mobile devices to autonomously perceive both short-term load fluctuations and long-term trends at edge nodes, without requiring global information or central coordination. This approach facilitates precise offloading decisions in complex, time-dependent environments, effectively handling uncertainties including unknown load patterns, sudden task surges, and heterogeneous resources. Comprehensive large-scale simulation experiments demonstrate the proposed method’s excellent robustness and generalization capabilities under varied resource configurations. Training convergence experiments indicate stable convergence after approximately 350 iterations, while high-concurrency scenarios (with up to 150 devices) show system performance fluctuations maintained within acceptable limits. The method achieves continuous optimization of key performance metrics, significantly reducing both task dropout rates and average processing latency, thereby offering a viable solution for efficient scheduling of latency-sensitive tasks. The effectiveness of the proposed approach assumes moderate-to-high edge computing capacity and periodic experience uploading from terminals, with terminals performing only lightweight decision-making. In extremely low-bandwidth environments, experience uploading may decelerate convergence, though local scheduling decisions remain executable without interruption. In future work, we plan to extend the proposed framework toward fully decentralized multi-agent reinforcement learning, where mobile devices can explicitly exchange local observations or learned representations to enable cooperative decision-making under partial observability. In addition, federated reinforcement learning will be explored to further enhance privacy preservation and scalability, allowing distributed agents to collaboratively improve policies without sharing raw data.

## Figures and Tables

**Table 1:** Comparison of Existing Task Offloading Approaches.

Method Category	Representative Works	Information Requirement	Scalability	Load Perception	Coordination Overhead
Heuristic-Based	[12,13,14,15]	Local observations	High	Single-scale (instantaneous)	None
Centralized DRL	[16,17,18]	Global state	Low	Single-scale (current state)	High (central coordinator)
Distributed DRL	[19,20,21,22,23]	Local observations	High	Single-scale (local features)	Low to Moderate
Prediction-Assisted	[24,25,26]	Historical data + current state	Moderate	Multi-scale (short-term only)	Moderate
**Proposed Method**	This paper	Local observations + historical load	**High**	**Multi-scale (short-term + long-term)**	**Low (distributed)**

**Table 2:** Task Dropout Rates for Each Algorithm Under Different Training Episodes.

Episode	No.OffL	R.OffL	PGOA	ULOOF	C-o	I-o	w/o-d	C-I-D3QN
0	0.50	0.22	0.21	0.17	0.13	0.12	0.11	0.11
250	0.50	0.22	0.21	0.17	0.09	0.08	0.06	0.05
500	0.50	0.22	0.21	0.17	0.07	0.07	0.04	0.03
750	0.50	0.22	0.21	0.17	0.06	0.06	0.04	0.03
1000	0.50	0.22	0.21	0.17	0.06	0.06	0.04	0.03

**Table 3:** Average Latency of Each Algorithm Under Different Training Episodes.

Episode	No.OffL	R.OffL	PGOA	ULOOF	C-o	I-o	w/o-d	C-I-D3QN
0	0.74	0.67	0.64	0.62	0.59	0.58	0.56	0.56
250	0.74	0.67	0.64	0.62	0.54	0.53	0.50	0.48
500	0.74	0.67	0.64	0.62	0.53	0.52	0.48	0.46
750	0.74	0.67	0.64	0.62	0.52	0.51	0.47	0.45
1000	0.74	0.67	0.64	0.62	0.52	0.51	0.47	0.45

**Table 4:** Steady-State Performance Comparison (mean ± std over 5 runs).

Method	Drop Rate	Avg Delay (s)
No.OffL	0.50±0.00	0.74±0.00
R.OffL	0.22±0.00	0.67±0.00
PGOA	0.21±0.00	0.64±0.00
ULOOF	0.17±0.00	0.62±0.00
CNN-only	0.06±0.006	0.52±0.008
Informer-only	0.06±0.005	0.51±0.007
w/o dueling	0.04±0.004	0.47±0.006
C-I-D3QN	0.03±0.004	0.45±0.007

**Table 5:** Task Drop Rate of Each Algorithm Under Different Task Arrival Probabilities.

Task Arrival	No.OffL	R.OffL	PGOA	ULOOF	C-o	I-o	w/o-d	C-I-D3QN
0.1	0.05	0.04	0.03	0.02	0.01	0.01	0.01	0.01
0.3	0.50	0.24	0.22	0.18	0.05	0.04	0.04	0.03
0.5	0.82	0.65	0.62	0.52	0.20	0.18	0.16	0.14
0.7	0.90	0.86	0.82	0.71	0.47	0.44	0.40	0.38
0.9	0.96	0.94	0.90	0.78	0.72	0.69	0.65	0.63

Note: The Task Arrival represents the Task Arrival Probability.

**Table 6:** Average Latency of Each Algorithm Under Different Task Arrival Probabilities.

Task Arrival	No.OffL	R.OffL	PGOA	ULOOF	C-o	I-o	w/o-d	C-I-D3QN
0.1	0.54	0.43	0.43	0.43	0.43	0.42	0.41	0.40
0.3	0.72	0.65	0.61	0.60	0.52	0.51	0.50	0.48
0.5	0.82	0.82	0.73	0.70	0.65	0.54	0.62	0.60
0.7	0.82	0.85	0.73	0.71	0.71	0.70	0.69	0.68
0.9	0.71	0.78	0.65	0.71	0.71	0.70	0.69	0.68

Note: The Task Arrival represents the Task Arrival Probability.

**Table 7:** Task Drop Rate of Each Algorithm Under Different Processing Capacity of Device.

Device CAP	No. OffL	R. OffL	PGOA	ULOOF	C-o	I-o	w/o-d	C-I-D3QN
1	0.93	0.21	0.20	0.205	0.13	0.11	0.09	0.08
2	0.67	0.21	0.20	0.18	0.10	0.08	0.07	0.05
3	0.35	0.21	0.18	0.11	0.07	0.06	0.04	0.01
4	0.12	0.21	0.10	0.08	0.04	0.03	0.02	0.01
5	0.03	0.21	0.06	0.06	0.02	0.01	0.01	0.01
6	0.01	0.21	0.04	0.04	0.01	0.01	0.01	0.01

Note: The Device CAP represents the processing capacity of device (unit: GHz).

**Table 8:** Task Drop Rate of Each Algorithm Under Different Processing Capacity of Edge.

Edge CAP	No.OffL	R.OffL	PGOA	ULOOF	C-o	I-o	w/o-d	C-I-D3QN
15	0.50	0.79	0.45	0.47	0.09	0.09	0.08	0.07
35	0.50	0.25	0.24	0.19	0.03	0.03	0.02	0.01
55	0.50	0.18	0.20	0.15	0.03	0.02	0.01	0.01
75	0.50	0.17	0.18	0.14	0.03	0.02	0.01	0.01

Note: The Edge CAP represents the processing capacity of edge (unit: GHz).

## Data Availability

Data are contained within the article. Further inquiries can be directed to the corresponding author.
